# A strategy for enrichment of claudins based on their affinity to *Clostridium perfringens *enterotoxin

**DOI:** 10.1186/1471-2199-10-61

**Published:** 2009-06-22

**Authors:** Dörte Lohrberg, Eberhard Krause, Michael Schümann, Jörg Piontek, Lars Winkler, Ingolf E Blasig, Reiner F Haseloff

**Affiliations:** 1 Leibniz-Institut für Molekulare Pharmakologie (FMP), Robert-Rössle-Str. 10, D-13125 Berlin, Germany

## Abstract

**Background:**

Claudins, a family of protein localized in tight junctions, are essential for the control of paracellular permeation in epithelia and endothelia. The interaction of several claudins with *Clostridium perfringens *enterotoxin (CPE) has been exploited for an affinity-based enrichment of CPE-binding claudins from lysates of normal rat cholangiocytes.

**Results:**

Immunoblotting and mass spectrometry (MS) experiments demonstrate strong enrichment of the CPE-binding claudins -3, -4 and -7, indicating specific association with glutathione-S-transferase (GST)-CPE_116–319 _fusion protein. In parallel, the co-elution of (non-CPE-binding) claudin-1 and claudin-5 was observed. The complete set of co-enriched proteins was identified by MS after electrophoretic separation. Relative mass spectrometric protein quantification with stable isotope labeling with amino acids in cell culture (SILAC) made it possible to discriminate specific binding from non-specific association to GST and/or matrix material.

**Conclusion:**

CPE_116–319 _provides an efficient tool for single step enrichment of different claudins from cell lysates. Numerous proteins were shown to be co-enriched with the CPE-binding claudins, but there are no indications (except for claudins -1 and -5) for an association with tight junctions.

## Background

The permeation of small molecules through the paracellular pathway in epithelial and endothelial cells is controlled by tight junctions, which constitute a continuous intercellular contact. The tight junction network, organized in strands, is formed by different proteins, among them *Zonula occludens *(ZO) proteins, claudins, occludin and junctional adhesion molecules. Although the regulation and the molecular composition of the tight junctions are far from being understood, it is generally accepted that claudins play a crucial role in tightening cell-cell contacts. The expression of claudins varies in different cell types and organs, and their protein-protein interactions which seal the paracellular space have been intensively discussed [1,2]. Different members of the claudin family, in particular claudin-3 and -4, are known to be receptor molecules of *Clostridium perfringens *enterotoxin (CPE) [3]; the COOH-terminal part of this 35 kDa protein has been shown to bind to the second extracellular loop of claudin-3 [4]. The minimum binding region of CPE has been narrowed down to the last 30 amino acids [5] and deletion of the six C-terminal amino acids abolishes claudin-binding activity [6].

The enrichment of proteins from a complex mixture is still a challenge, in particular when the target structures are at low abundance and/or are integral membrane proteins. Moreover, one of the major concerns in affinity-based enrichment strategies is non-specific binding, which must be diminished or at least identified [7-9]. In addition to co-immunoprecipitation techniques [10], there is increasing interest in applying recombinant proteins [11] or protein domains [12] for selectively enriching proteins; there are, however, only a few examples directed at tight junction proteins. A co-immunoprecipitation approach (based on the binding of tight junction proteins to atypical protein kinase C zeta) has been applied for purification of the tight junction complex [13]. The interaction of ZO-1 with α-actinin-4 has been demonstrated by a pull-down assay utilizing immobilization of recombinant PDZ domains of this tight junction protein [14].

The investigations presented here are aimed at extracting members of the claudin protein family from cell lysates, using a pull-down assay based on the affinity of CPE_116–319 _to the second extracellular loop of several claudins. The binding proteins are eluted, electrophoretically separated and identified by mass spectrometry (MS). Additionally, in combination with stable isotope labeling with amino acids in cell culture (SILAC) [15], it is investigated whether this approach allows the identification of proteins specifically associated with claudins and, consequently, with the tight junction complex.

## Methods

### Expression and purification of recombinant bait protein

The plasmid bearing *Clostridium perfringens *enterotoxin (CPE)-cDNA (pPROEX-1-CPE) was kindly provided by Y. Horiguchi (Osaka, Japan). To produce glutathione S-transferase (GST)-CPE, the DNA-sequence of CPE_116–319 _was subcloned into *Eco*RI/*Sal*I sites in the pGEX-4T-1 vector.

GST-CPE_116–319 _fusion protein and GST were expressed in *E. coli *(BL21). After induction, bacteria were grown to an optical density of 0.7 ± 0.1 (at 600 nm) and were harvested by centrifugation for 10 min at 3200 × g. The pellets were resuspended in lysis buffer containing 1% Triton X-100, 0.1 mM phenylmethylsulphonyl fluoride, 1 mM ethylenediaminetetraacetic acid (EDTA) and protease inhibitor cocktail (Sigma-Aldrich, Taufkirchen, Germany) in phosphate-buffered saline (PBS) and underwent two passages in an EmulsiFlex-C3 homogenizer (Avestin Europe GmbH, Mannheim, Germany). The insoluble cell debris was removed by centrifugation for 1 h at 40000 × g. For purification of the recombinant proteins, the clarified supernatants were loaded onto columns containing glutathione-agarose under gravity flow. The resin was rinsed twice with washing buffer (1% Triton X-100 in PBS). GST-CPE_116–319 _and GST were eluted from the resin with elution buffer (10 mM reduced glutathione, 50 mM Tris/HCl, pH 9.5). The samples were dialyzed against PBS. Protein concentrations were determined using the 2-D Quant Kit (GE Healthcare, Freiburg, Germany).

### Preparation of epithelial cell lysates

Normal rat cholangiocytes (NRC), a gift of N.F. LaRusso, Rochester, MN/USA, were cultured in 75 cm^2 ^rat tail collagen-coated cell culture flasks in DMEM/HAM's F12 medium (Biochrom, Berlin, Germany) with 5% CO_2 _in air [16]. Arginine- and lysine-deficient medium (Biochrom) was used for SILAC experiments, where one cell population was supplemented with [^12^C_6_]arginine and [^12^C_6_]lysine, whereas another cell population was grown in medium containing [^13^C_6_]arginine and [^13^C_6_]lysine. Cells were grown to confluence, washed twice with ice-cold PBS to remove serum proteins and scraped in ice-cold PBS. The cell suspensions were centrifuged for 10 min at 300 × g; the pellets were frozen in liquid nitrogen and stored at -80°C.

NRC cell pellets corresponding to four 75 cm^2 ^cell culture flasks, prepared under either labeled or unlabeled conditions, were resuspended in 1 ml lysis buffer (1% Triton X-100, 50 mM Tris/HCl, pH 7.4, 150 mM NaCl, and Complete protease inhibitors, EDTA-free, Roche Diagnostics GmbH, Mannheim, Germany). Cells were homogenized using a syringe and a 24-gauge needle. After incubation on ice for 30 min, the cell extract was cleared by centrifugation for 10 min at 10000 × g.

### Affinity purification

Glutathione-sepharose™ 4B beads (GE Healthcare, Freiburg, Germany) were washed with cold PBS before use. Equal amounts of GST-CPE_116–319 _(this construct was found in preceding experiments to exert a stronger association to the second extracellular loop of claudin-3 as compared to CPE_290–319_) or GST were rotated with beads for 1 h at 4°C. After three washing steps with PBS containing 1% Triton X-100, the beads loaded with GST-CPE_116–319 _were incubated with the lysate of labeled cells for 2 h at 4°C. As a control, beads were loaded with GST and rotated in the lysate of unlabeled cells for the same period of time. In addition, a corresponding inverse experiment (^13^C-labeling of GST fraction) was carried out. Non-specifically bound proteins were diminished by rinsing the beads three times with a washing buffer (twice with 1% and once with 0.1% Triton X-100 in PBS). Bound proteins were eluted with 200 μl of elution buffer (10 mM reduced glutathione, 50 mM Tris/HCl, pH 9.5) in two steps. Beads were incubated in elution buffer for 10 min at 4°C. Both eluates were pooled.

### Immunoblotting

Proteins were separated by 15% sodium dodecyl sulfate polyacrylamide gel electrophoresis (SDS-PAGE) and transferred to a nitrocellulose membrane. After blocking with 5% nonfat milk in Tris-buffered saline with Tween-20 (TBST, containing 10 mM Tris/HCl, pH 7.4, 150 mM NaCl, 0.05% Tween-20), the membranes were washed three times in TBST. Membranes were incubated at 4°C overnight with monoclonal antibodies to either claudin-1, -3, -4, -5, or -7 (Invitrogen/Zymed Laboratories, Karlsruhe, Germany), diluted in TBST. After washing three times in TBST, the immunoblots were incubated with horseradish peroxidase-conjugated anti-rabbit (claudin-4, anti-mouse) secondary antibody (Invitrogen) for 1 h at room temperature. The membranes were washed for at least 30 min, exchanging TBST every 10 min, and signals were visualized by horseradish peroxidase-dependent chemiluminescence (GE Healthcare, Freiburg, Germany). For sphingosine kinase 2 (SphK2) immunoprecipitation, 2 μg of the primary monoclonal antibody (Santa Cruz Biotechnology, Heidelberg, Germany) were added to 50 μl of NRC lysate (500 μg total protein) and incubated for 1 h at 4°C. Subsequently, 10 μl of resuspended protein A/G PLUS agarose (Santa Cruz Biotechnology, Heidelberg, Germany) were added and incubated for 1 h at 4°C. As a control, 10 μl of protein A/G PLUS agarose were incubated with 50 μl of NRC lysate for 1 h at 4°C. Immunoprecipitates were collected by centrifugation at 300 × g for 1 min at 4°C. The pellet was washed twice with 1% and once with 0.1% Triton X-100 in PBS, each time repeating the centrifugation step. After the final wash, the pellet was resuspended in 20 μl electrophoresis sample buffer (2% SDS, 10% glycerol, 0.01% bromophenol blue and 0.1 M dithiothreitol in 50 mM Tris/HCl, pH 6.8). Samples were heated for 5 min at 40°C and analyzed by SDS-PAGE and immunoblotting.

### Identification and quantitation of proteins by mass spectrometry

The eluates from the GST-CPE_116–319 _and the control GST pull-down were combined at a ratio of 1:1, resolved by SDS-PAGE (4%–18%) and stained with Coomassie Brilliant Blue G-250. Excised gel slices were washed with 50% (v/v) acetonitrile in 50 mM ammonium bicarbonate, shrunk by dehydration in acetonitrile, and dried in a vacuum centrifuge. The dried pieces were reswollen in 15 μl of 50 mM ammonium bicarbonate containing 60 ng trypsin (sequencing grade, Promega, Mannheim, Germany). After 16 h incubation at 37°C, 15 μl of 0.3% trifluoroacetic acid (TFA) in acetonitrile were added, and the separated supernatant was dried under vacuum. For nanoLC-MS/MS, the samples were dissolved in 6 μl of 0.1% (v/v) TFA, 5% (v/v) acetonitrile in water.

Tandem MS experiments were performed on a quadrupole orthogonal acceleration time-of-flight mass spectrometer Q-Tof Ultima (Micromass, Manchester, UK), equipped with a liquid chromatography system (CapLC, Waters GmbH, Eschborn, Germany), as previously described [17]. In brief, LC-separations were performed on a capillary column (Atlantis dC_18_, 3 μm, 100 Å, 150 mm × 75 μm i.d., Waters) at an eluent flow rate of 200 nl/min, using an acetonitrile gradient in 0.1% formic acid (v/v). MS/MS data were acquired in a data-dependent mode, using MS survey scanning followed by MS/MS of the most abundant peak. Data analysis was performed with MassLynx version 4.0 software (Micromass-Waters).

The processed MS/MS spectra and MASCOT server (version 2.0, Matrix Science Ltd, London, UK) were used to search in-house against the SwissProt protein database. The maximum of two missed cleavages was allowed and the mass tolerance of precursor and sequence ions was set to 100 ppm and 0.1 Da, respectively. Acrylamide modification of cysteine, methionine oxidation, ^13^C_6_-arginine and ^13^C_6_-lysine were considered as possible modifications. A protein was accepted as identified if the total MASCOT score was greater than the significance threshold and at least two peptides appeared for the first time in the report and these peptides were first ranking. Protein identifications were performed at a < 1% false positive rate as established by decoy database search strategy.

Quantitation was based on calculations of isotope intensity ratios of at least two arginine- or lysine-containing tryptic peptides that were identified by MS/MS with a score above the MASCOT identity threshold. Additional criteria were that no interfering mass peaks were observed, that the peptide appeared for the first time in the report and that it was a first ranking peptide.

## Results and Discussion

Tight junction proteins, their homophilic and heterophilic interactions and their regulation, are of outstanding importance for the function of many organs. The present study is devoted to the claudin protein family, as its members play a decisive role in providing paracellular tightness. Based on their affinity to CPE_116–319_, enrichment of different claudins from cell lysates has been accomplished. Co-enriched proteins have been identified and their potential interaction with claudins assessed.

Several epithelial cell lines were tested initially, and normal rat cholangiocytes were selected for subsequent experiments, as higher levels of CPE-binding claudins -3, -4 and -7 were detected by immunoblotting when compared to the human colon carcinoma cell line Caco-2. However, it should be mentioned that the MS identification of the proteins eluted from GST-CPE_116–319 _after incubation with Caco-2 lysates indicated that claudin-2 and claudin-6 also accumulate in this fraction (cf. additional file 1, table_a.pdf – Identification of claudins in Caco-2 and NRC cells).

In preceding experiments, different protocols were tested with respect to the enrichment of claudins. A huge number of non-specifically bound proteins were identified by mass spectrometry in a protein fraction obtained by a co-immunoprecipitation experiment using a monoclonal claudin-3 antibody (data not shown). Moreover, the data indicated lower relative enrichment of claudin-3 than with the CPE-based approach and the lack of any claudin different from claudin-3. Thus, another advantage of enrichment using CPE is its high affinity to several members of the claudin family.

**Figure 1:**
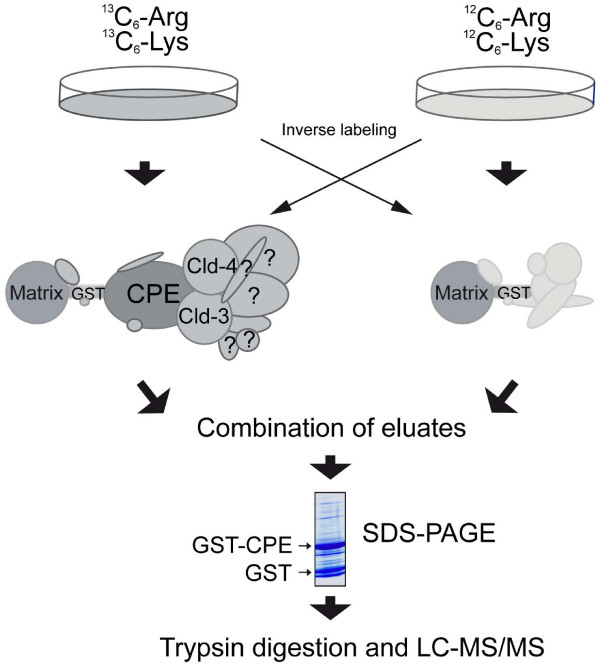
**Strategy for affinity purification using GST- CPE_116–319 _as "bait"**. For affinity purification, one cell population is grown in medium containing [^12^C]arginine and [^12^C]lysine, whereas the other is grown in medium supplemented with [^13^C]arginine and [^13^C]lysine. Bound proteins of both pull-down fractions are combined and separated by SDS-PAGE. After tryptic in-gel digestion, proteins are identified by LC-MS/MS, and the ratios of their abundance are quantified from MS peak intensities.

The scheme shown in Fig. 1 specifies the protocol applied for affinity chromatographic enrichment of claudins and subsequent MS identification of co-eluted proteins. As initial experiments revealed relatively strong non-specific binding to various carrier materials, a protein labeling strategy (SILAC) was employed in order to define proteins non-specifically bound to GST and/or matrix. Cell lysates containing labeled proteins were added to CPE_116–319_-loaded GST fusion protein, whereas the non-labeled protein fraction was added to the unloaded control (or vice versa in an inverse labeling experiment). In NRC cells, the degree of ^13^C-Arg and ^13^C-Lys labeling was 90% (which was corrected for in the values given below).

**Figure 2:**
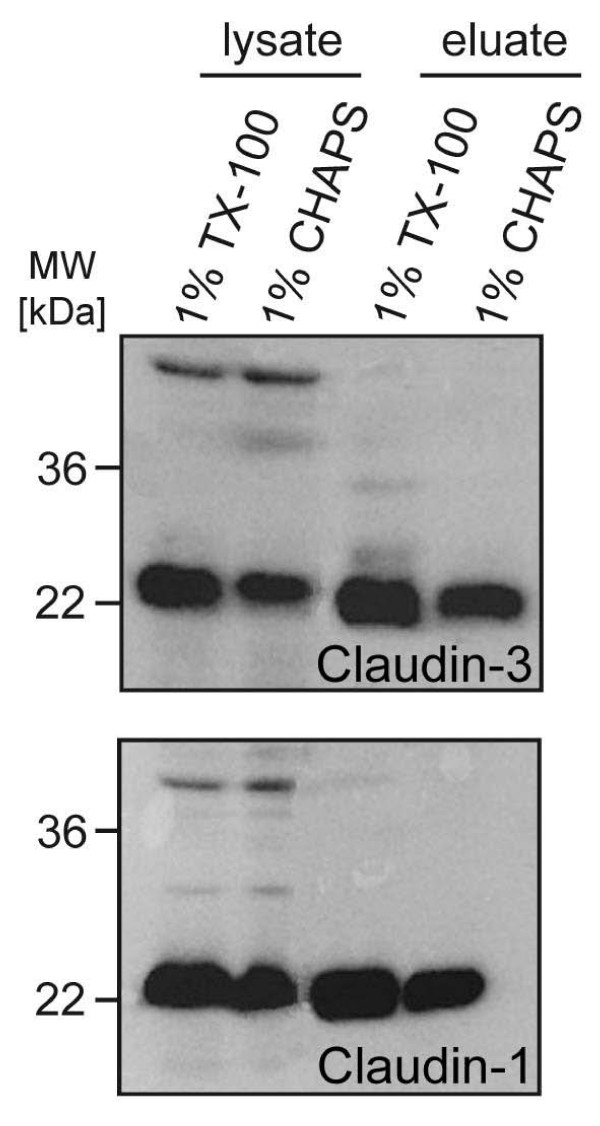
**Solubilization of claudins by different detergents**. Immunoblots against claudin-3 (upper panel) and claudin-1 (lower panel) of protein fractions from NRC cells (lysate and GST-CPE_116–319_eluate) solubilized by Triton X-100 or CHAPS.

The experimental procedures were optimized with respect to the amount of claudins enriched in the eluate fractions. Different detergents were tested to improve the solubilization of claudins in the lysate and eluate fractions; Fig. 2 gives a comparison of the recovery of claudin-1 and claudin-3 in lysis buffers containing Triton X-100 and 3-[(3-cholamidopropyl)dimethylammonio]-1-propanesulfonate (CHAPS). Triton X-100 was selected for further experiments, as higher yields of the claudins were found both in the lysate- and in the GST-CPE_116–319 _eluate fraction. Corresponding experimental conditions have also been successfully used in a study on the self-association of tight junction proteins [18].

**Figure 3:**
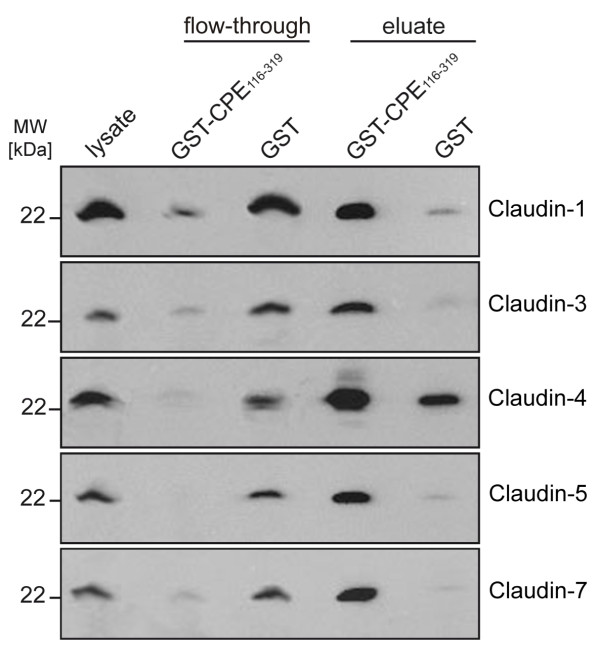
**Claudin enrichment in GST-CPE_116–319 _eluate fraction**. Immunoblots against claudin-1, claudin-3, claudin-4, claudin-5 and claudin-7 of protein fractions obtained from NRC cells after GST-CPE_116–319 _affinity purification; lane 1, lysate (positive control); lane 2, flow-through of GST-CPE_116–319 _fraction; lane 3, flow-through of control fraction (GST only); lane 4, eluate of GST-CPE_116–319 _fraction; lane 5, eluate of control fraction.

Immunoblots against claudins of the different flow-through and eluate fractions clearly demonstrate the strong enrichment of claudins -1, -3, -4, -5 and -7 in the GST-CPE_116–319 _eluate fraction (Fig. 3). The blots of the eluate fractions of the GST controls show a faint signal originating from claudin-1 and a stronger signal of claudin-4, possibly indicating weak association of these claudins with GST and/or matrix material.

The C-terminal part of CPE (amino acids 290–319) is sufficient for interactions with claudin-3 and claudin-4 [5]. Neither claudin-3 nor claudin-4 were detected by immunoblotting in a control affinity enrichment applying a truncated (CPE_194–309_) construct (cf. additional file 2, figure_a.pdf – Association of claudin -3 and claudin-4 with truncated CPE constructs). It has been reported [19] that tight junction strands gradually disintegrate and disappear from the cell surface after treatment with the toxin. However, despite its high affinity interaction with claudins, opening of the tight junctions by CPE treatment needs hours [20] which corresponds with our observation (data not shown) that a significantly lower amount of claudin-3 is precipited with CPE_116–319 _when the construct is incubated with intact cell monolayers instead of lysates.

For MS analysis, the eluted GST and GST-CPE_116–319 _fractions were mixed at a 1:1 ratio, separated by SDS-PAGE, and in-gel digested. For the identified proteins, the degree of binding specificity is given by the value R, defined here as the mean of the MS peak intensity ratios (^13^C-labeled/unlabeled or vice versa for inverse labeling) of peptides originating from CPE_116–319_- and control fraction. Table 1 lists the proteins identified in at least two independent experiments with R ≥ 3 (additional information based on [21] and [22] is included). In accordance with the immunoblotting data, the highest ratios were obtained for the claudins. High association constants (K_a _> 5 × 10^7 ^M^-1^) with CPE have been published for claudin-3 and -4 [19], as well as for claudin-7 [4]. Claudin-1 and claudin-5 (expressed at lower levels in NRC cells) do not bind to CPE according to the literature [4,19]. The unambiguous identification of these claudins by immunoblotting (claudin-1 also by MS) could be explained either by binding to CPE_116–319 _or by a linkage to other CPE-binding proteins. There are indeed literature data reporting interaction of claudins -1 and -3 in co-expression studies [23,24], and the association of claudin-3 and claudin-5 was demonstrated in transfected cell lines and human airway epithelium [24]. Additional experiments performed in either claudin-3- or claudin-5-transfected HEK293 cells revealed an association of claudin-3, but not claudin-5, to CPE_116–319 _under the experimental conditions applied here (data not shown). Ongoing investigations will clarify the molecular basis of the specific enrichment of claudin-1 and -5 in the GST-CPE_116–319 _fraction.

**Table 1:** Proteins enriched in GST-CPE_116–319 _fraction as detected by mass spectrometry

**AN**	**Protein**	**MW_app_**	**MW**	**SC**	**N**	R	**comment**
Q63400	Claudin-3	20000	23269	298	6	**11.9**	tight junction
O35054	Claudin-4	20000	22150	170	3	**11.0**	tight junction
Q9Z1L1	Claudin-7	20000	22416	157	2	**11.0**	tight junction
P56745	Claudin-1	20000	22850	171	3	**10.2**	tight junction
Q8CFI7	DNA-directed RNA polymerase II subunit RPB2	120000	133911	1911	42	**7.7**	nuclear
P60898	DNA-directed RNA polymerase II subunit RPB9	10000	14532	234	3	**7.7**	nuclear
Q9ERU9	Ran-binding protein 2^#^	*130000*	341091	579	14	**7.6**	nuclear
P97760	DNA-directed RNA polymerase II subunit RPB3	30000	31311	470	7	**6.2**	nuclear, cytosolic
Q99M87	DnaJ homolog subfamily A member 3	36000	52443	90	2	**6.1**	mitochondrial
O15514	DNA-directed RNA polymerase II subunit RPB4	14000	16311	134	2	**6.0**	nuclear
P62832	60S ribosomal protein L23	14000	14865	296	7	**5.5**	ribosomal
Q9R112	Sulfide:quinone oxidoreductase	38000	50340	916	18	**4.9**	mitochondrial
Q5RK28	Normal mucosa of esophagus-specific gene 1 protein	5000	9598	143	3	**4.2**	nuclear
Q9D6M3	Mitochondrial glutamate carrier 1	27000	34670	153	4	**4.1**	mitochondrial
Q5I0H3	Small ubiquitin-related modifier 1 (SUMO-1)	*85000*	11557	147	3	**4.1**	nuclear, cytosolic
P46061	Ran GTPase-activating protein 1	*85000*	63616	506	9	**4.0**	nuclear, cytosolic
P29147	D-beta-hydroxybutyrate dehydrogenase	25000	38202	129	3	**3.9**	mitochondrial
Q62425	NADH dehydrogenase 1 alpha subcomplex	7000	9327	234	5	**3.3**	mitochondrial
P13437	3-ketoacyl-CoA thiolase	40000	41871	589	14	**3.3**	mitochondrial
Q9JIA7	Sphingosine kinase 2	64000	65618	109	2	**3.3**	cytosolic
Q8BH59	Calcium-binding mitochondrial carrier protein Aralar1	62000	74570	394	8	**3.2**	mitochondrial
Q66X93	Staphylococcal nuclease domain-containing protein 1	99000	101952	795	18	**3.1**	nuclear
Q8N163	KIAA 1967	110000	102902	613	12	**3.1**	nuclear
Q5I0E6	RNA polymerase II-associated protein 2	64000	67892	140	3	**3.1**	not assigned^1^
Q925I1	ATPase family AAA domain-containing protein 3	53000	66742	565	12	**3.0**	mitochondrial

Each protein was identified in at least two of three independent experiments (including one experiment applying inverse labeling). AN, UniProtKB/SwissProt protein accession number; MW_app_, apparent molecular weight [Da] according to position in gel; MW, molecular weight [Da] according to UniProtKB/SwissProt; SC, protein (Mascot™) score (highest value of individual experiment); N, highest number of peptides identified in individual experiment; R, ratio between MS peak intensities of peptides originating from GST-CPE_116–319 _and GST elution fractions; comment, cellular component according to UniProtKB/SwissProt (^1^nuclear/cytosolic according to [22]); ^#^, protein fragment

In addition to heterologous claudin-claudin interactions, claudins associate with ZO-1 [25] and possibly tetraspanins [26]. However, neither of these proteins was found by MS (ZO-1 also immunoblot) in the GST-CPE_116–319 _fraction. Except for the claudins, the vast majority of proteins found to be enriched in the GST-CPE_116–319 _fraction are of nuclear or mitochondrial origin. Among the proteins identified to co-elute with the claudins, the most specific association with the CPE construct was observed for DNA-directed RNA polymerase II. Interestingly, at least one other protein was identified which can be bound to this polymerase (RNA polymerase II-associated protein 2 [27]). However, the interaction of this nuclear protein with the CPE complex is, although specific, thought to be a false positive (not claudin-related), since it is likely to be favored by the disintegration of cellular structures given in cell lysates. This type of interaction must also be assumed for other proteins, *e.g*., mitochondrial proteins, which are strictly separated *in vivo *from the plasma membrane. The function(s) known so far for Ran-binding protein 2 (RBP2) and RanGTPase-activating protein 1 (RanGAP1) are related to nucleocytoplasmic transport, also suggesting a false positive interaction. Both RBP2 and RanGAP1 are substrates of post-translational modification by small ubiquitin-like modifier 1 (SUMO-1) [28], which was also identified. Moreover, RanGAP1 and SUMO-1 were found at an apparent molecular mass of 85 kDa in the same gel slice as the only proteins with R > 3, indicating that RanGAP1 is the protein sumoylated here.

**Figure 4:**
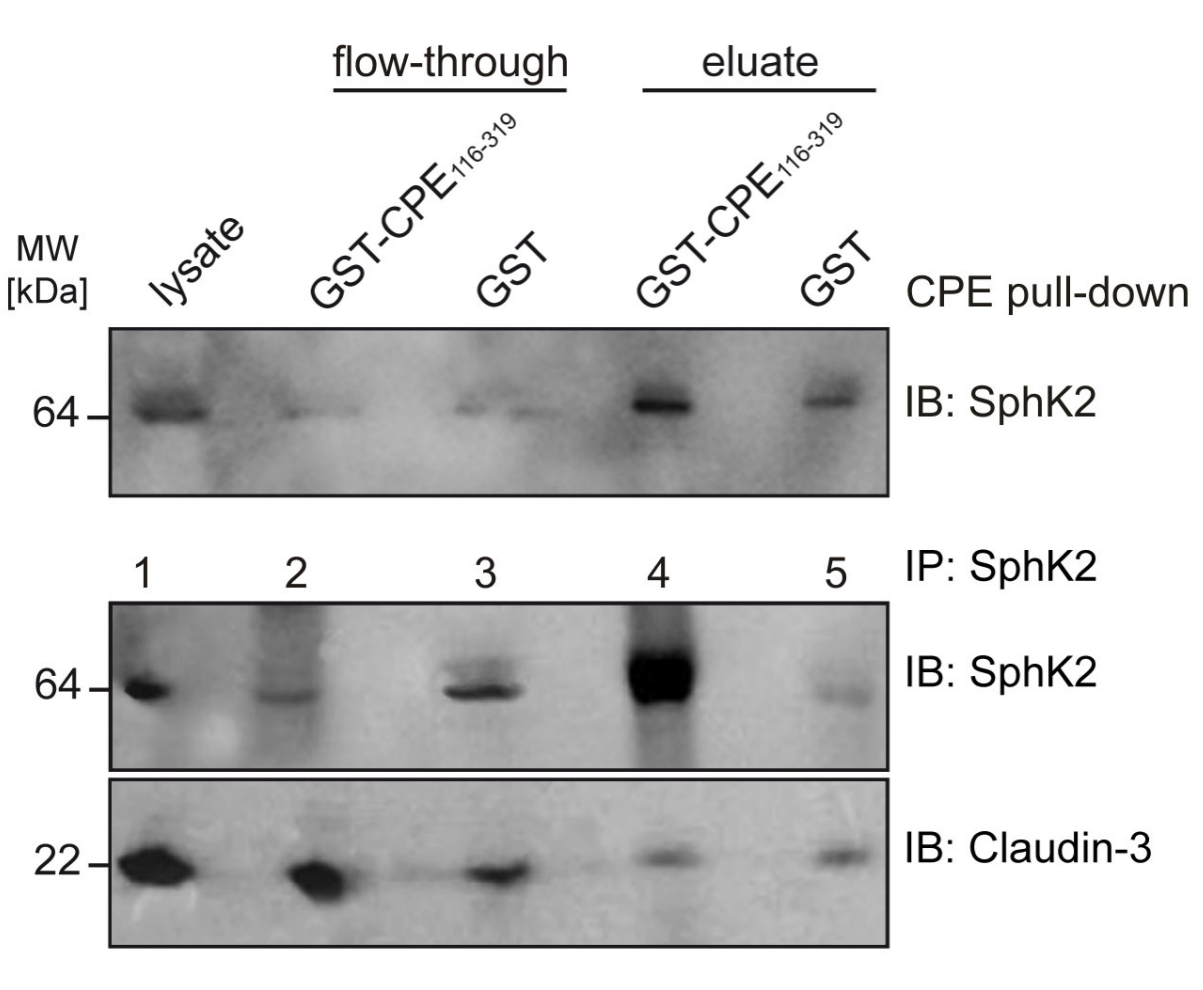
**Sphingosine kinase enrichment in GST-CPE_116–319 _eluate fraction and sphingosine kinase immunoprecipitation**. Immunoblot (IB) against sphingosine kinase 2 (SphK2) of protein fractions after GST-CPE_116–319 _affinity purification (upper panel); immunoblots against SphK2 (middle panel) and claudin-3 (lower panel) of protein fractions obtained by SphK2 immunoprecipation (IP); lane 1, lysate of NRC cells; lane 2, flow-through of IP fraction; lane 3, flow-through of control fraction (protein A/G plus-Agarose); lane 4, eluate of IP fraction; lane 5, eluate of control fraction.

The cytosolic localization of sphingosine kinase 2 could facilitate association with intracellular parts of claudins (although this protein has recently been found to localize to the nucleus too [29]). Moreover, a connection exists between sphingosine kinase 2 and the junctional complex: sphingosine 1-phosphate (the product of sphingosine phosphorylation by sphingosine kinases) induces in endothelial cells the formation of ZO-1 complexes with cortactin and α-catenin, which regulate chemotactic response and barrier integrity, respectively [30]. Additional experiments have therefore been performed to check for a possible interaction of claudins with sphingosine kinase 2. The immunoblot given in Fig. 4 (upper panel) demonstrates enrichment of SphK2 in the GST-CPE_116–319 _fraction, although a lower amount of the protein was also found in the respective GST control. Co-immunoprecipation using a monoclonal antibody directed against SphK2 revealed strong enrichment of SphK2 in the eluate fraction (Fig. 4, middle panel). However, co-immunoprecipitation of claudins with SphK2 was not detectable, neither for claudin-3 (Fig. 4, lower panel) nor for claudins -1, -4 or -7 (data not shown). Moreover, immunofluorescence staining did not provide any evidence for co-localization of SphK2 and claudins in NRC cells (data not shown).

## Conclusion

The present study introduces a new approach directed at enriching the CPE-binding claudins. The proteins interacting specifically with GST-CPE_116–319 _include not only claudins -3, -4 and -7 (which are known to bind to the toxin), but also claudins -1 and -5, pointing at a possible indirect heterologous association of these tight junction proteins. Different proteins (and potentially also protein complexes) were shown to co-elute from the GST-CPE_116–319 _complex, but there is no indication that any of these proteins plays a role with respect to tight junctions or cell-cell contacts. Simultaneous enrichment of numerous claudins may nevertheless provide a useful tool applicable to further investigations devoted to these tight junctional proteins.

## Authors' contributions

DL performed cell culture work, biochemical experiments/data analysis and participated in drafting the manuscript. EK and MS designed and performed the mass spectrometry analysis, EK participated in drafting the manuscript. JP participated in designing the biochemical experiments, immunofluorescence experiments and drafting the manuscript; LW participated in performing the biochemical experiments. IEB participated in the design of the study and in drafting the manuscript. RFH conceived of the study, participated in its design and coordination, in the analysis of the mass spectrometry data and in drafting the manuscript. All authors read and approved the final manuscript.

## Supplementary Material

Additional file 1 **Identification of claudins in Caco-2 and NRC cells**. A table is provided which gives an overview of the different claudins detected in NRC and Caco-2 cells by immunoblotting and mass spectrometry. Click here for file

Additional file 2 **Association of claudin-3 and claudin-4 with truncated CPE constructs**. Immunoblots against claudin-3 and claudin-4 obtained from NRC pull-down fractions of CPE_194–309 _and CPE_194–319 _are provided which demonstrate that these claudins bind to CPE_194–319 _but not to the construct truncated by 10 amino acids from the C-terminal end. Click here for file
