# Supplementary material for: A strategy for enrichment of claudins based on their affinity to Clostridium perfringens enterotoxin
Source: BMC Mol Biol. 2009 Jun 22;10:61. doi: 10.1186/1471-2199-10-61 (PMC2713237; doi:10.1186/1471-2199-10-61)
Supplement: Additional file 1 — Identification of claudins in Caco-2 and NRC cells. A table is provided which gives an overview of the different claudins detected in NRC and Caco-2 cells by immunoblotting and mass spectrometry. [file 1471-2199-10-61-S1.pdf]

Table A: Identification of claudins in Caco-2 and NRC cells

	Immunoblot cell lysate		Mass spectrometry $CPE_{116-319}$-enriched fraction	
	Caco-2	NRC	Caco-2#	NRC
Claudin-1	×	×	–	×
Claudin-2	×	–	×	–
Claudin-3	×*	×	×	×
Claudin-4	×	×	×	×
Claudin-5	–	×*	–	–
Claudin-6	×	–	×	–
Claudin-7	×*	×	–	×

# Experiments in Caco-2 cells were accomplished without ^{13}C-labeling; $CPE_{116-319}$- and control (GST) fractions were analyzed separately. No member of the claudin protein family was identified in the control fraction.

* Weak signal.

Antibodies used: rabbit anti-claudin-1, -2, -3, -5, -7, mouse anti-claudin-4, all obtained from Invitrogen, Karlsruhe/Germany;
goat anti-claudin-6 obtained from Santa Cruz Biotechnology, Heidelberg/Germany.
