# Supplementary material for: A strategy for enrichment of claudins based on their affinity to Clostridium perfringens enterotoxin
Source: BMC Mol Biol. 2009 Jun 22;10:61. doi: 10.1186/1471-2199-10-61 (PMC2713237; doi:10.1186/1471-2199-10-61)
Supplement: Additional file 2 — Association of claudin-3 and claudin-4 with truncated CPE constructs. Immunoblots against claudin-3 and claudin-4 obtained from NRC pull-down fractions of CPE194–309 and CPE194–319 are provided which demonstrate that these claudins bind to CPE194–319 but not to the construct truncated by 10 amino acids from the C-terminal end. [file 1471-2199-10-61-S2.pdf]

Association of claudin-3 and claudin-4 with truncated CPE constructs

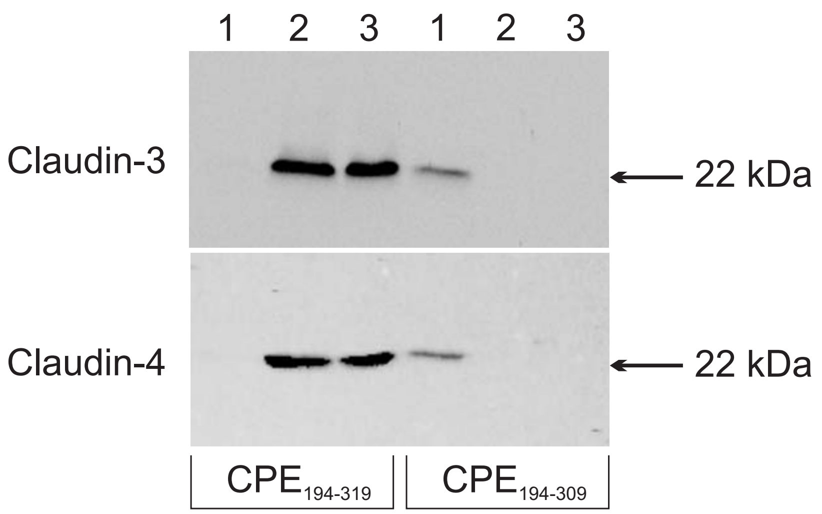

Figure A: Immunoblots of CPE pull-down fractions of NRC cell lysates demonstrating that $CPE_{194-319}$, but not $CPE_{194-309}$ binds to claudin-3 and claudin-4:
1, flow-through fraction (8% of total fraction);
2, elution fraction obtained using elution buffer (80% of total fraction);
3, elution fraction obtained by subsequent elution using electrophoresis sample buffer (80% of total fraction); buffer components as described in main manuscript.
